# Reasons for encounter and health problems managed by general practitioners in the rural areas of Beijing, China: A cross-sectional study

**DOI:** 10.1371/journal.pone.0190036

**Published:** 2017-12-21

**Authors:** Yanli Liu, Chao Chen, Guanghui Jin, Yali Zhao, Lifen Chen, Juan Du, Xiaoqin Lu

**Affiliations:** 1 Department of General Practice, School of General Practice and Continuing Education, Capital Medical University, Beijing, P.R. China; 2 Department of Education, Xuanwu Hospital, Capital Medical University, Beijing, P.R. China; Australian National University, AUSTRALIA

## Abstract

**Objective:**

The purpose of this study was to describe the patients’ reasons for encounter (RFE) and health problems managed by general practitioners (GPs) in the rural areas of Beijing to provide evidences for health services planning and GPs training.

**Methods:**

This study was conducted at 14 community health service centers (CHSCs) in 6 suburban districts of Beijing, using a multistage sampling method. A total of 100 GPs was selected from the study sites. A self-designed data collection form was developed on the basis of Subjective-Objective-Assessment-Plan (SOAP), including patient characteristics, RFEs, health problems, interventions, and consultation length. Each GP recorded and coded their 100 consecutive patients’ RFEs and health problems with the International Classification of Primary Care, 2nd version (ICPC-2). Descriptive statistics were employed to describe the distribution of RFE and health problems. Student t-test and analysis of variance were used to compare the differences of mean number of RFE or health problems per encounter by patient characteristics.

**Results:**

A total of 10,000 patient encounters with 13,705 RFEs and 15,460 health problems were recorded. The RFEs and health problems were mainly distributed in respiratory, circulatory, musculoskeletal, endocrine, metabolic and nutritional, and digestive systems. Cough and hypertension were the most common RFE and health problem, respectively. With increased ages, the mean number of RFEs decreased and the mean number of health problems increased. Patients with Beijing medical insurance had less RFEs and more health problems than those in other cities (p<0.001). Patients who had visited the CHSC previously and signed contracts with the GP team had more health problems than those who had not (p<0.001).

**Conclusions:**

These findings present a view of patients’ demands and work contents of GPs in Beijing rural areas and can provide reference for health services planning and GPs training.

## Introduction

A good primary care infrastructure is positively associated with health outcomes and negatively associated with costs on health care [1]. And there can’t be a good primary care system without general practice. General practice was introduced into China in 1980s. To speed up the development of general practice and improve the quality of primary care services, a series of policies including finance support, personnel training, pharmaceutical system, and health insurance were established by the Chinese government in the past 2 decades [2–6]. In the past decade, the primary care network was mostly developed around China. Till the end of 2012, 3,3562 CHSC were established across the nation [7]. A CHSC is a primary care institution in where GPs can provide health services for local residents [8]. In the CHSC, the GP provides services in a team approach with nurses, and preventive medicine professionals. Patients are encouraged to sign a contract with one of the GP teams in CHSC. The team is responsible for providing continuous care and additional services for patients who have signed contracts [9].

Currently, China is in the transition process of health reform and primary health care plays a leading role in it. For policy and the professional education purposes, it is important to understand patients’ needs and health problems in the primary care population. Patients’ demands can be reflected by their reasons for encounter (RFEs) which refers to the reasons why a patient enter the health care system. Diagnoses or health problems refer to provider’s assessment of patient’s health problems and reflect provider’s view. It was demonstrated that RFEs were the main element to impact the patient’s perspective on the diagnostic process and subsequent interventions [10].

The International Classification of Primary Care (ICPC), developed by the WONCA International Classification Committee (WICC) in 1987, is a useful tool to record patient RFEs and health problems in primary care. It includes three important elements which could be provided in primary care system: reasons for encounter (RFEs), diagnoses or problems, and process of care. It has 17 chapters based on body systems, with one additional chapter for psychological problems and one for social problems [11]. Each chapter is divided into seven components. Component 1 codes symptoms and complaints, while Component 7 codes diseases. A RFE can be either a symptom (Component 1) or a disease (Component 7). A health problem usually has a disease label diagnostic title. However, if a disease-label diagnostic is not appropriate, ICPC recommends the use of the symptom itself as a diagnostic [10]. Components 2–6 deal with interventions and can be used to code an RFE which is presented as a request for an intervention. It is argued that documenting and coding the RFEs with ICPC can improve the quality of primary care data [10].

As the first city introducing general practice into Mainland China, Beijing is also one of the pilot provinces for health reform. Moreover, GPs training for rural areas had been initiated since 2012 in several cities of China. Beijing was among the first group of cities implementing this training program. Therefore, knowledge of the general practice in pilot cities like Beijing can provide reference for other provinces.

Several studies had recorded RFEs and health problems through ICPC in urban CHSCs of Beijing [12–13], while little is known about that in rural areas. Previous research suggested that rural patients and GPs were different from their non-rural counterparts [14]. The aim of this study was to investigate patients’ RFE and health problems managed by GPs in rural areas of Beijing to provide evidence for developing policies on health services planning and GPs training in rural areas.

## Method

### Ethics statement

This study was approved by the Ethical Committee of Capital Medical University, Beijing, China. Written informed consents were obtained from all participants involved in this study. For participants under the age of eighteen, written informed consents were obtained from their guardians. All participants were ensured that their information were kept confidential and only be used in this research.

### Subjects selected and data acquisition

Multistage sampling method was adopted in this study. As the Capital of China, Beijing has 6 urban districts and 10 suburban districts. According to the Provisions on the Division of Urban and Rural Areas proposed by the National Bureau of Statistic, suburban districts are subdivided into towns and rural areas[15]. On the first stage, 6 of 10 suburban districts in Beijing were selected randomly as study sites. On the second stage, 14 rural CHSCs in these study sites were selected by purposive sampling method with the following criteria: (1) located in the rural areas, (2) stable amounts of patients, and (3) available for this study. Experts of general practice were consulted before purposive sampling. On the third stage, 100 GPs who met the following inclusion criteria were invited to participate in this survey: (1) worked in this center for 5 years or above, (2) obtained the medical license of practicing doctor, (3) obtained certificates of general practice training, and (4) are now undertaking the diagnosis and treatment work in clinical practice.

Before the investigation, a research team was established, including two research experts of general practice, two GPs and three postgraduates of general practice. After discussion, the research team developed a data collection form specifically for this study which was based on the Subjective-Objective-Assessment-Plan (SOAP), an approach of recording electronic patient record (EMR). The contents included (1) patients’ socio-demographic data, (2) up to 4 reasons for encounter (RFEs) and codes, (3) diagnoses/health problems and codes, (4) interventions, and (5) the consultation length. Each participating GP was required to record the details of consecutive 100 patients’ encounters during or after the consultation with the data collection forms.

The GPs were trained before the beginning of the study in terms of the purpose and contents of this survey, specifications of recording forms, and the principles of ICPC-2 coding. Training courses were conducted in the Capital Medical University. One of the research experts in our team (XL) who participated in the translation of ICPC-2 from English into Chinese in 2000, was responsible for introducing the concept and code principles of RFEs and health problems using ICPC-2 in the training courses. A book on clarification of the inclusion and exclusion for codes was distributed to each GP. The research expert also trained two postgraduates as central coders, who assumed the responsibility of supervising the whole research process. The research experts were consulted when there were any difficulties in coding.

The investigation began on December 8, 2014. The main purpose of this study was to obtain an as comprehensive spectrum of the patients’ RFEs and health problems as possible. Therefore, if a patient returned for the same problem within the same week, only the first encounter was recorded. Completed forms were collected and checked by two postgraduates in the research team. For forms with missing data, the GPs were required to record a new patient’s encounter to replace it. Eventually, the survey ended on January 27, 2015.

### Statistical analysis

Epidata 3.0 was used to establish the database and double-entry was performed by two postgraduates in our research team. All data analyses were carried out in SPSS for Windows 22.0. Descriptive statistics were employed to describe the number, distribution and rank of patients’ RFE and health problems. Student t-test and analysis of variance were used to compare the differences of mean number of RFE or health problems per encounter according to patient characteristics.

## Results

### The characteristics of participating GPs

Of the 100 participating GPs, 59% were males and their mean age was 40.4±6.46 (ranging from 27 to 65) years. Most of the participating GPs (68%) obtained the education level of university. Their mean length working in rural CHSCs was 18.3±6.88 (ranging from 27 to 65) years. Detailed information on characteristics of the GPs is presented in Table 1.

**Table 1:** Characteristics of the participating GPs.

GPs characteristic	N/%
**Sex**	
Male	41
Female	59
**Age group**	
<35 years	19
35–44 years	57
45–54 years	22
55+ years	2
**Final education level**	
University	68
Senior college	29
Junior college or secondary school	3
**Years in current rural CHSCs**	
5–9 years	15
10–19 years	39
≥ 20 years	46

### Distribution of RFEs and health problems

A total of 10,000 patients encounters were recorded by 100 GPs. There were 13,705 RFEs (1.37 per encounter) and 15,460 health problems (1.55 per encounter) from 10,000 encounters. The average consultation length of each encounter was 8.1±6.98 minutes. The distribution of RFEs and health problems according to the17 chapters of the ICPC-2 are shown in Table 2. “R: Respiratory”, “K: Circulatory”, L: “Musculoskeletal”, “T: Endocrine, metabolic and nutritional”, and “D: Digestive”, were among the top five chapters for both RFEs and health problems.

**Table 2:** The frequencies of RFE and health problems, according to the chapters in the ICPC- 2.

ICPC-2 chapter	RFE frequencies	RFE (%) n = 13,705	ICPC-2 chapter	Health problem frequencies	Health problems (%) n = 15,460
**R respiratory**	3498	47.9	**K circulatory**	5563	36.0
**K circulatory**	2028	27.8	**R respiratory**	3846	24.9
**L musculoskeletal**	1065	14.6	**T endocrine, metabolic and nutritional**	1995	12.9
**T endocrine, metabolic and nutritional**	880	12.1	**L musculoskeletal**	1418	9.2
**D digestive**	806	11.0	**D digestive**	1014	6.6
**A general and unspecified**	546	7.5	**A general and unspecified**	476	3.1
**N neurological**	463	6.4	**U urology**	212	1.4
**U urology**	186	2.6	**X female genital system**	201	1.3
**X female genital system and breast**	161	2.2	**S skin**	178	1.2
**P psychological**	117	1.6	**P psychological**	148	1.0
**S skin**	79	1.1	**N neurological**	126	0.8
**F eye**	79	1.1	**F eye**	110	0.7
**B blood, blood forming organs, lymphatics, spleen**	32	0.4	**Y male genital system**	80	0.5
**Y male genital system**	32	0.4	**B blood, blood forming organs, lymphatics, spleen**	51	0.3
**H ear**	22	0.3	**W pregnancy, childbirth, family planning**	23	0.2
**Z social problems**	36	0.1	**H ear**	19	0.1
**W pregnancy, childbirth, family planning**	3	0.0	**Z social problems**	0	0
**Total**	13,705	100	**Total**	15,460	100

### Top 30 recorded RFEs and health problems managed by rural GPs

Tables 3 and 4 present the top 30 RFEs and health problems recorded, respectively. In this part, we referred to BEACH (Bettering the Evaluation and Care of Health) program of Australia [16]. Code groups were used to analyze the reasons for encounter and problems managed, which means some RFEs and health problems include multiple ICPC-2 codes. For example, “Abdominal pain” includes D01, Abdominal pain/cramps, general, D02, Abdominal pain, epigastric, and D06, Abdominal pain, localized, other. “Hypertension” includes K86, uncomplicated hypertension, and K87, complicated hypertension. In this study, we distinguished the prescription refill and follow up encounter. The former referred to prescription as the only reason for encounter. When patients visited the CHSCs with requests for other intervention for an already existing health problem except for prescription, then the RFE of this encounter was defined as follow up encounter.

**Table 3:** The top 30 RFEs in descending order of frequency.

Order	ICPC-code	Patient reason for encounter	Frequencies	RFEs (%) n = 13,705	Cumulative %
1	R05	**Cough**	1,841	13.4	13.4
2	K50	**Prescription refill for hypertension**	1685	12.3	25.7
3	R21	**Throat symptom/complaint**	1,101	8.0	33.7
4	T50	**Prescription refill for diabetes**	785	5.7	39.4
5	R07	**Sneezing/nasal congestion**	772	5.7	45.1
6	A03	**Fever**	440	3.2	48.3
7	N17	**Vertigo/dizziness**	388	2.8	51.1
**8**	R25	**Sputum/phlegm abnormal**	385	2.8	53.9
**9**	K50	**Prescription refill for ischaemic heart disease**	249	1.8	55.7
**10**	D01, D02, D06	Abdominal pain*	231	1.7	57.4
**11**	T50	**Prescription refill for lipid disorder**	189	1.4	58.8
**12**	D03	**Heartburn**	186	1.4	60.2
**13**	L02, L03, L86	Back complaint*	177	1.3	61.5
**14**	P06	**Sleep disorders**	149	1.1	62.6
**15**	N01	**Headache**	143	1.0	63.6
**16**	L15	**Knee symptom/complaint**	142	1.0	64.6
**17**	-30, -31	Check up- all*	124	0.9	65.5
**18**	D08	**Flatulence/gas/belching**	113	0.8	66.3
**19**	U02	**Urinary frequency/urgency**	107	0.8	67.1
**20**	K04	**Palpitations/awareness of heart**	106	0.8	67.9
**21**	K50	**Prescription refill for cerebrovascular disease**	96	0.7	68.6
**22**	L14	**Leg/thigh symptom/complaint**	84	0.6	69.2
**23**	U01	**Dysuria/painful urination**	83	0.6	69.8
**24**	D18	**Change in faeces/bowel movement**	81	0.6	70.4
**25**	L20	**Joint symptom/complaint NOS**	81	0.6	71
**26**	K02	**Pressure/tightness of heart**	77	0.6	71.6
**27**	K63	**Follow-up encounter for Hypertension**	74	0.5	72.1
**28**	K63	Follow-up encounter for Ischaemic heart disease*	71	0.5	72.6
**29**	L04	**Chest symptom/complaint**	71	0.5	73.1
**30**	R03	**Wheezing**	67	0.5	73.6
**Total**	-	-	10,098	73.6	-

* Include multiple ICPC-2 codes.

**Table 4:** Top 30 health problems in descending order of frequency.

Order	ICPC-code	Health problems	Frequencies	Health problems (%) n = 15,460	Cumulative %
**1**	K86, K87	Hypertension*	3529	22.8	22.8
**2**	R74	**Upper respiratory infection, acute**	2287	14.8	37.6
**3**	T89, T90, W85	Diabetes-all*	1511	9.8	47.4
**4**	K74, K76	Ischaemic heart disease*	1167	7.6	55.0
**5**	K89, K90, K91	Cerebrovascular disease*	677	4.4	59.4
**6**	L89, L90, L91	Osteoarthrosis*	604	3.9	63.3
**7**	T93	**Lipid disorder**	421	2.7	66.0
**8**	A90	**No disease**	418	2.7	68.7
**9**	D87	**Stomach function disorder**	414	2.7	71.4
**10**	R77	**Laryngitis/tracheitis, acute**	387	2.5	73.9
**11**	R78	**Acute bronchitis/bronchiolitis**	362	2.3	76.2
**12**	L84, L86	Back syndrome*	236	1.5	77.7
**13**	R76	**Tonsillitis, acute**	202	1.3	79.0
**14**	R83	**Respiratory infection, other**	201	1.3	80.3
**15**	R79	**Chronic bronchitis**	147	1.0	81.3
**16**	L83	**Neck syndrome**	130	0.8	82.1
**17**	L95	**Osteoporosis**	127	0.8	82.9
**18**	D84	**Oesophagus disease**	122	0.8	83.7
**20**	U70, U71	Urinary tract infection*	121	0.8	84.5
**19**	D85, D86	Peptic ulcer*	118	0.8	85.3
**21**	D12	**Constipations**	110	0.7	86.0
**22**	P06	**Sleep disturbance**	88	0.6	86.6
**23**	A80	**Trauma/injury NOS**	79	0.5	87.1
**24**	R80	**Influenza**	70	0.5	87.6
**25**	Y85	**Benign prostatic hypertrophy**	70	0.5	88.1
**26**	X88	**Fibrocystic disease breast**	57	0.4	88.5
**27**	R81	**Pneumonia**	56	0.4	88.9
**28**	D82	**Teeth/gum disease**	52	0.3	89.2
**29**	X84	**Vaginitis/vulvitis NOS**	52	0.3	89.5
**30**	L87	**Bursitis/tendinitis/synovitis NOS**	47	0.3	89.8
**Total**	**-**	-	13, 862	89.8	-

* Include multiple ICPC-2 codes.

The top 30 RFEs accounted for 73.6% of all RFEs, most of which were symptom descriptions such as cough, fever, or throat symptoms/complaint. Cough was the most common RFE, accounting for 13.4% of the total RFEs. The results showed that prescription refill for chronic diseases accounted for a high proportion.

Table 4 presented the top 30 health problems, which accounted for 89.8% of all health problems. Chronic diseases, including hypertension, diabetes, ischaemic heart disease, and cerebrovascular diseases, accounted for almost half of total health problems (44.6%). Acute upper respiratory infection was the second health problem.

### The RFEs of patients with common health problems

For patients with only one health problem, acute hypertension, upper respiratory infection, and diabetes were the top three diagnoses. To understand how RFEs related to health problems, we described the top 5 RFEs of patients with these three problems. Table 5 showed that symptoms were main RFEs in acute upper respiratory infection, while reasons like prescription refill and follow up were dominant in hypertension and diabetes.

**Table 5:** Top 5 RFEs for patients with top 3 common health problems.

**Hypertension**	N	%
K50 Prescription refill for hypertension	765	66.8
K63 Follow-up encounter for hypertension	175	14.8
N17 Vertigo/dizziness	107	9.0
K25 Fear of hypertension	13	1.1
K29 Cardiovascular symptom/complaint, other	11	0.9
Total top 5	1071	92.6
Total	1184	100
**Acute upper respiratory infection**	N	%
R05 Cough	581	36.5
R21 Throat symptom/complaint	327	20.5
R07 Sneezing/nasal congestion	297	18.6
A03 Fever	182	11.4
R50 Request for medications	85	5.3
Total top 5	1472	92.3
Total	1593	100
**Diabetes**	N	%
T50 Prescription refill for diabetes	369	78.5
Follow-up encounter for diabetes	68	14.5
T01 Excessive thirst	13	2.8
T02 Excessive appetite	5	1.1
U02 Urinary frequency/urgency	4	0.9
Total top 5	459	97.7
Total	470	100

### Number of RFEs and health problems per encounter and their distribution for patient characteristics

Table 6 shows the mean number of RFEs and health problems per encounter. The majority of patients (70.3%) presented the CHSCs with only one RFE. Patients who had only one health problem accounted for 59.1%.

**Table 6:** Numbers of RFEs and health problems per encounter.

Number of RFEs	Number of encounters	%	Number of health problems	Number of encounters	%
**No RFE**	0	0	**No disease**	418	41.8
**One RFE**	7029	70.3	**One problem**	5914	59.1
**Two RFEs**	2303	23.0	**Two problems**	2424	24.3
**Three RFEs**	602	6.0	**Three problems**	866	8.7
**Four RFEs**	66	0.7	**Four problems or above**	378	3.8
**Total**	10, 000	100	**Total**	10, 000	100

Note: RFEs—reasons for encounter.

Table 7 presents the difference of mean number of RFEs and health problems per encounter classified by patient characteristics. With the increase of ages, the mean number of RFEs decreased and the mean number of health problems increased. Patients aged 14 or below had more RFEs and less health problems than other age groups. Patients with medical insurance in Beijing had less RFEs and more health problems than those in other cities (p<0.001). Patients for the first time to this center had more RFEs and less health problems than patients who had visited to this center previously (p<0.001). Patients who had signed contracts with the GPs team had more health problems than those who had not (p<0.001).

**Table 7:** Mean number of RFE and health problems per encounter by patient characteristics.

Patient Characteristics	N	Mean number of RFE (95% CI)	P value	Mean number of health problems (95% CI)	P value
**Sex**^(a)^			0.515		0.618
Male	4746	1.32 (1.31–1.34)		1.51(1.48)	
Female	5235	1.32 (1.30–1.34)		1.50(1.54)	
**Age groups**^(a)^			0.000*		0.000*
0–14 years	307	1.66 (1.57–1.75)		1.11 (1.06–1.16)^§^	
15–24 years	221	1.44 (1.36–1.51)^†^		1.01 (0.94–1.08)^§^	
25–44 years	1802	1.36 (1.37–1.39)^†^		1.24 (1.20–1.27)	
45–59 years	3613	1.28 (1.27–1.30)^‡^		1.48 (1.46–1.51)	
60 years or above	4014	1.30 (1.29–1.32)^‡^		1.70 (1.67–1.73)	
**Medical insurance location**^(a)^			0.001*		0.000*
Beijing	9354	1.32(1.31–1.33)		1.51 (1.49–1.53)	
Other provinces	230	1.46(1.38–1.55)		1.16(1.07–1.25)	
**Is it the first time to this center?**^(a)^			0.000*		0.000*
Yes	689	1.42(1.37–1.46)		1.17 (1.12–1.23)	
No	9046	1.32(1.30–1.33)		1.53 (1.51–1.55)	
**Whether sign contracts with the GP team?**^(a)^			0.131		0.000*
Yes	5892	1.33(1.31–1.34)		1.66 (1.63–1.68)	
No	3807	1.31(1.29–1.33)		1.27 (1.24–1.29)	

^(a)^The missing data was removed.

*There was statistically significant difference.

^†^There was no statistically significant difference between the 15–24 years group and the 25–44 years group, p>0.05.

^‡^There was no statistically significant difference between the 45–59 years group and the 60 years or above group, p>0.05.

^§^There was no statistically significant difference between the 0–14 years group and the 15–24 years group, p>0.05. Unless otherwise indicated, there was statistically significant difference for mean number of RFE and health problem per encounter between each two age groups.

## Discussion

To optimize primary health care and improve the service quality, it is necessary to know whom we are seeing and why they see us [17]. Health providers in general practice should not only be directed at health problems but also be responsive to patients’ need [18]. In this study, both RFEs and health problems managed were recorded and coded to reflect the needs of rural population as well as the work contents of GPs in rural areas of Beijing.

### The distribution of RFEs and health problems

The results showed that GPs in rural areas served patients from all age groups. The RFEs and health problems were distributed in almost all organs and body systems. The health problems managed by GPs included both acute and chronic problems. These reflected the comprehensive characteristic of general practice. However, psychological problems in this study were not as common as other populations. A study conducted in a tertiary hospital found that psychological problem accounted for 12.5% and depression was the fifth diagnosis [19]. Studies conducted in primary care of other countries also found a higher proportion of psychological problems [20–23]. One of the explanations might be the lack of knowledge on psychological symptoms and problems for patients and GP in rural areas. In ICPC-2, chronic alcohol abuse and tobacco abuse belong to psychological chapter. However, almost no one came the CHSC for these reasons and no related diagnoses were made by GPs. Moreover, it might be related to different cultural backgrounds. In Chinese culture, many patients with psychological or psychiatric problems are still associated with stigma, especially in relatively poor rural areas. Even though patients have psychological symptoms, they may not go to consult the GPs. This finding implied that the whole-person care of general practice was not achieved in rural areas of Beijing in terms of mental health. It revealed that GPs should recognize and pay attention to patients’ potential psychological complaints. Training on psychological problems should be strengthened in the future.

### The common RFEs and health problems

The similarities in common RFEs and health problems were found between our study and other populations, especially in RFEs [23–26]. Examples included cough, fever, throat symptoms/complaints, headache, and back complaints. Although cough was the most common RFE, we found that hypertension was the most common health problem. This was not contradictory. In this study, we collected up to 4 RFEs presented by patients and all health problems diagnosed by GPs. The health problems included both existing illnesses and new diagnoses. For example, some patients visit the CHSC for cough or other reasons, but he might be diagnosed with hypertension previously.

Like other populations, chronic non-communicable diseases, e.g. hypertension, diabetes, were common problems managed by rural GPs [25–26]. Corresponding to a high proportion of chronic diseases, prescription refill was a very common RFE in this study. This finding was consistent with a study conducted in urban areas of Beijing [27]. It revealed a special phenomenon that GPs both in urban and rural areas spent a large amount of time on prescription refill for patients. There may be some reasons for this phenomenon. On the one hand, CHSCs have implemented standardized chronic disease management since recent years and most patients with chronic diseases can be managed within general practice by GPs [28]. On the other hand, according to the Regulation of Prescription Management issued by the Ministry of Health and policies of medical insurance, to improve the quality of prescription and ensure the safety of patients, patients with chronic diseases have to renew medications for chronic diseases every two weeks or one month [29].

The mix nature of acute onset diseases was found in our study. Acute onset diseases in the primary care setting consist of two categories: 1) major acute diseases that threaten life expectancy and lasting functioning (for example: MI; pulmonary embolism), and 2) self-limiting illness (for example: cough; URTI; gastro-enteritis; low back pain). For acute diseases, early diagnosis and intervention are important. While for self-limiting illness, the emphasis is on health education, and a restraint approach, avoiding over-prescribing (antibiotics, pain medication) and medicalization.

When prescription or follow-up become the main content of GPs’ daily work, we may concern that their treatment of acute onset diseases may be hindered or limited. On the one hand, the consultation length will be greatly shortened. Jin GH found that the media consultation length was only 2.0 minutes in CHSC. Even for patients with symptoms, the consultation length was only 3.0 minutes. Moreover, he found that the physical examination and history taking provided by GPs were reduced [27]. This might affect the service quality. On the other hand, when GPs spent most of their time on prescription and follow-up for chronic diseases, they may lose their ability to deal with acute onset diseases.

We further described the top 5 RFEs of common health problems. The results presented us the variation RFEs between various health problems. It confirmed the conclusion that symptoms were the main RFEs in URTI, while prescription refill and follow up were main RFEs in chronic diseases like hypertension and diabetes. These findings were of great significance. Because patients visiting the CHSC for the same health problem might have different reasons to consult, this may lead to different response and management from the GPs [18]. We found that although a high prevalence of chronic illnesses existed in this population, follow up encounter was much fewer than prescription refill in management of these diseases. It is worthy of our attention that GPs should not only repeat prescription for patients with chronic diseases. They have more important roles in the management of these diseases, such as monitoring disease progress and offering the best treatment regime to these patients.

### The difference in number of RFEs and health problems among patients with different characteristics

Another finding was that patients of different characteristics had differences in demands for health care and co-morbid problems. With the age increases, patients have less RFEs but more health problems. It was consistent with previous study that older patients had more health problems per visit than younger ones [30]. It suggested that co-morbid problems were more common in older patients.

We also found that patients without Beijing medical insurance had more RFEs but less health problems than those who had Beijing medical insurance. It might be related to the health care seeking behavior. A study found that the ratio of health care seeking behavior among migrants without Beijing medical insurance was lower than Beijing local residents [31]. Although the coverage rate of medical insurance is high in China, it is nearly impossible to transfer insurance relations between different cities. Patients without Beijing medical insurance have to pay the medical expense at their own when visit the community health centers in Beijing, which may result in more barrier to access health care even though they have more demands. Therefore, patients without Beijing medical insurance should be paid more attention in the future policy making process. For example, making the medical insurance interchangeable among different provinces and cities may be a solution to this problem.

Patients who had visited their primary care center previously and signed contracts with GPs team tended to have more health problems than those not. Two reasons can be used to explain these results. On the one hand, GPs are more familiar with the health situations of the patients they managed and those who visited them frequently. On the other hand, patients with multiple health problems returned to see the GPs more frequently than patients with fewer problems. It revealed the importance of continuous care provided by GPs for patients with multiple health problems.

## Strengths and limitations

China is in the process of health reform focusing on primary care. Many provinces of China, including Beijing, are implementing the GPs training programs in rural areas. This study presented the situation of general practice in rural areas of Beijing, which could provide important evidence for policy making and GPs training.

However, there were limitations in this study. First, limited by the conditions, purposive sampling was used when selecting rural CHSCs, which may have lead to bias. However, the sample selection strictly followed the inclusion criteria and 6 out of 10 suburban districts in Beijing were selected randomly. Therefore, we think this study could represent the work status of GPs in rural areas of Beijing. Second, this survey was conducted in winter season and the short duration of this investigation could not provide a longitudinal data. A high proportion of URTI may be related to the season to some extent. To reduce this bias, we plan to collect data of other seasons in the future study.

## Conclusion

The current study presented patients’ reasons for encounter and health problems managed by GPs in rural areas. GPs in rural areas of Beijing managed a range of health problems almost distributed in all body system and all age groups, which reflected the comprehensive care provided by GPs in some degree. However, psychological problems were not as common as other populations, which is worthy of the attention of GPs. Chronic diseases were common health problems among this population. Except for repeat prescription, GPs play more important roles in management of these diseases, such as monitoring disease progress and offering the best treatment regime to these patients.

As Chinese government is implementing the primary care reform, the data is helpful to understand the patient needs and work contents of GPs in rural areas as well as provide more evidence for the development of primary care.

## Supporting information

S1 File Date set on 100 patient encounters of a GP. (SAV)
